# Universal Glycosyltransferase Continuous Assay for Uniform Kinetics and Inhibition Database Development and Mechanistic Studies Illustrated on ST3GAL1, C1GALT1, and FUT1

**DOI:** 10.1021/acsomega.4c00485

**Published:** 2024-04-05

**Authors:** Abdullateef Nashed, Kevin J. Naidoo

**Affiliations:** † Scientific Computing Research Unit, University of Cape Town, PD Hahn Building, Rondebosch 7701, South Africa; ‡ Department of Chemistry, University of Cape Town, PD Hahn Building, Rondebosch 7701, South Africa

## Abstract

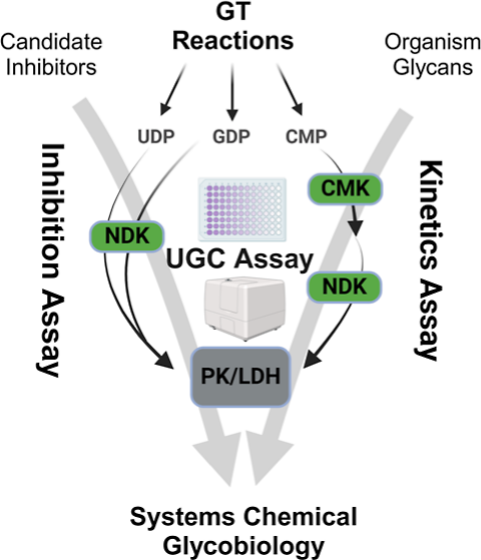

Chemical systems glycobiology requires experimental and computational tools to make possible big data analytics benefiting genomics and proteomics. The impediment to tool development is that the nature of glycan construction and mutation is not template driven but rests on cooperative glycosyltransferase (GT) catalytic synthesis. What is needed is the collation of kinetics and inhibition data in a standardized form to make possible analytics of glycan and glycoconjugate synthesis, mechanism extraction, and pattern recognition. Currently, kinetics assays in use for GTs are not universal in processing nucleoside phosphate UDP, GDP, and CMP donor-based glycosylation reactions due to limitations in accuracy and large substrate volume requirements. Here we present a universal glycosyltransferase continuous (UGC) assay able to measure the declining concentration of the NADH reporter molecule through fluorescence spectrophotometry and, therefore, determine reaction rate parameters. The development and parametrization of the assay is based on coupling the nucleotide released from GT reactions with pyruvate kinase, via nucleoside diphosphate kinase (NDK) in the case of NDP-based donor reactions. In the case of CMP-based reactions, the coupling is carried out via another kinase, cytidylate kinase in combination with NDK, which phosphorylates CMP to CDP, then CDP to CTP. Following this, we conduct kinetics and inhibition assay studies on the UDP, GDP, and CMP-based glycosylation reactions, specifically C1GAlT1, FUT1, and ST3GAL1, to represent each class of donor, respectively. The accuracy of calculating initial rates using the continuous assay compared to end point (noncontinuous) assays is demonstrated for the three classes of GTs. The previously identified natural product soyasaponin1 inhibitor was used as a model to demonstrate the application of the UGC assay as a standardized inhibition assay for GTs. We show that the dose response of ST3GAL1 to a serial dilution of Soyasaponin1 has time-dependent inhibition. This brings into question previous inhibition findings, arrived at using an end point assay, that have selected a seemingly random time point to measure inhibition. Consequently, using standardized *K*_m_ values taken from the UGC assay study, ST3GAL1 was shown to be the most responsive enzyme to soyasaponin1 inhibition, followed by FUT1, then C1GALT1 with IC_50_ values of 37, 52, and 886 μM respectively

## 1 Introduction

The structures of complex carbohydrates, termed glycans, underpin their many cellular roles. In living organisms, glycans covalently bonded to proteins, peptides, and lipids (glycoconjugates) are essential to cellular functions from energy metabolism to cell signaling. The functions of glycans are carried out either directly or by altering the properties of their conjugates. These alterations are determined by the structural properties of the glycans, which, in turn, are determined by the action of a combination of several types of glycosyltransferases (GTs). While it is known that the differential expression of GT genes is important to the development of diseases and can be used to classify cancer,^1^ the roles of the networks of cooperation between GTs in these diseases are not understood. Much of the work in health-related glycobiology to date has focused on signature glycans, however, the systems function of GTs in the living organisms has largely been ignored and has only now begun to be addressed.^2^ Building a systems model of glycan construction through GTs requires consistent kinetics and inhibition data to map out the network pathways that can be used in algorithmic paradigms such as Metabolic Control Analysis.^3−5^ Further, the key to measuring a GT’s role within a metabolic network is inhibitory control. However, paradoxically there are no clinically effective inhibitors of human and more generally mammalian GTs, partly because accurate assays and metabolite-specific enzyme kinetic parameters (*k*_cat_ and *K*_m_) are not readily available, which are the major prerequisite for accurate inhibitor screening parametrization. Inhibitors currently in use show class promiscuity, making testing in animal models impossible as they induce metabolic feedback loops, resulting in a global, family-wide shutdown of sialyltransferases (STs) and remodeling of cell-surface glycans.^6^ The synthetic challenges are formidable and as such only moderately successful attempts at GT inhibitors have been possible, all of which rely on donor frames.^7^

Unlike DNA, RNA and protein syntheses that are template driven, the architecture of each glycan in the human glycome is a result of GT expression ensembles from a possible 250 GTs that dynamically coordinate in their individual catalysis of their specific glycosylation reactions.

**Figure 1:**
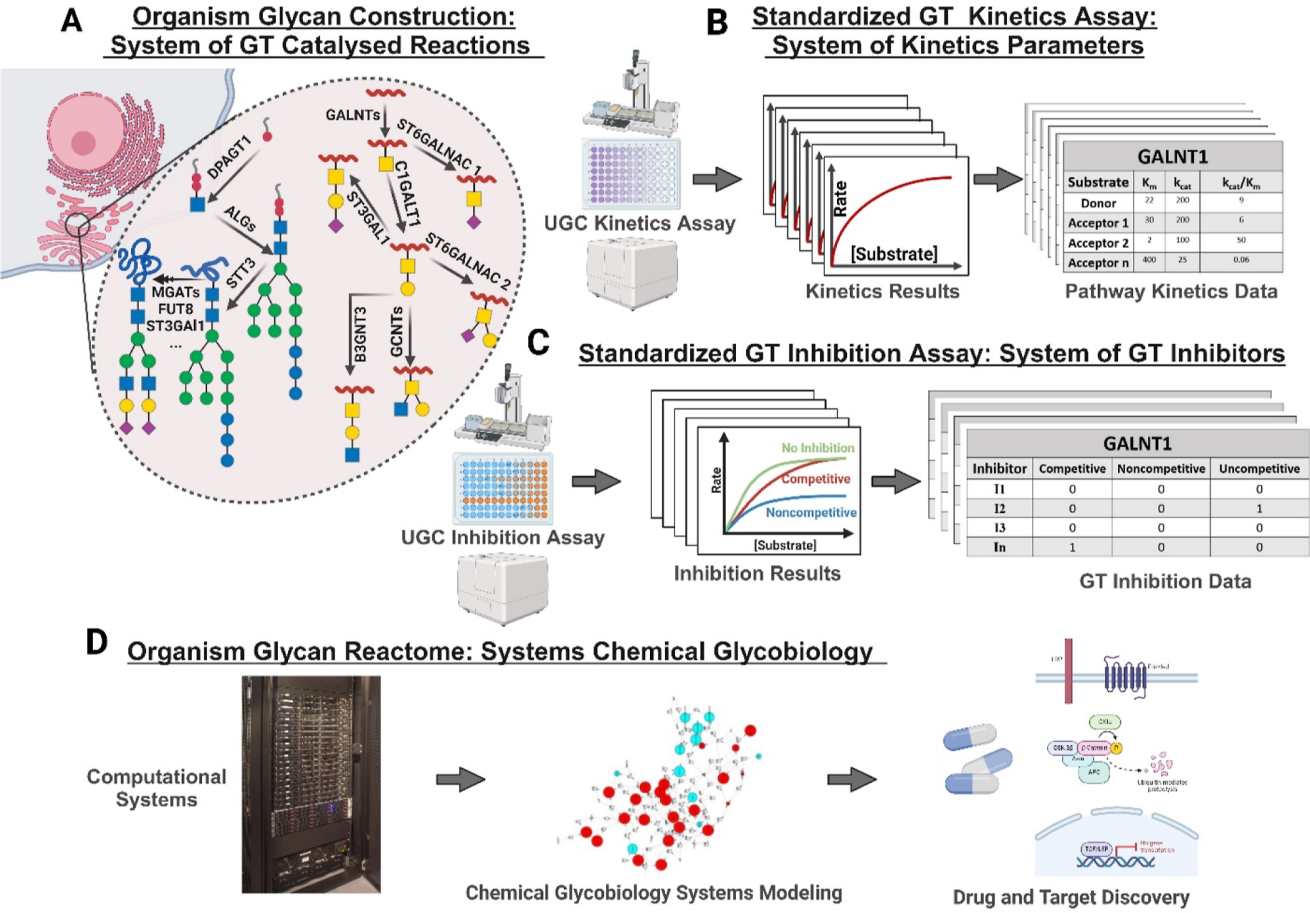
Systems chemical glycobiology data from standardized assay for model building. (A) Complexity of glycosylation pathways and glycan construction in organisms. (B) Transferable kinetics parameters for enzyme networks using standard assays. (C) Transferable inhibition parameters for enzyme networks using standard assays. (D) Data source for computational model building of chemical glycobiology networks.

A systems chemical biology model requires standardized kinetics and inhibitory parametric data for each GT catalyzing the reactions that construct varied substrates in the glycome (Figure 1A). To emulate the genomic and proteomic big data analytics successes in chemical glycobiology, it is best to employ a universal assay that is able to deliver standardized reproducible kinetic (Figure 1B) and inhibition (Figure 1C) data for each substrate family of GTs. Here, we describe the development of a universal glycosyltransferase continuous (UGC) assay that meets this challenge (Figure 1). We have developed a visual data analytics platform called CytoCopasi^8^ for systems chemical biology. Chemical glycobiology models can be built with UGC assay data and employed in CytoCopasi to simulate the effects of each GT on metabolites that contribute to a phenotype (Figure 1D).

**Figure 2:**
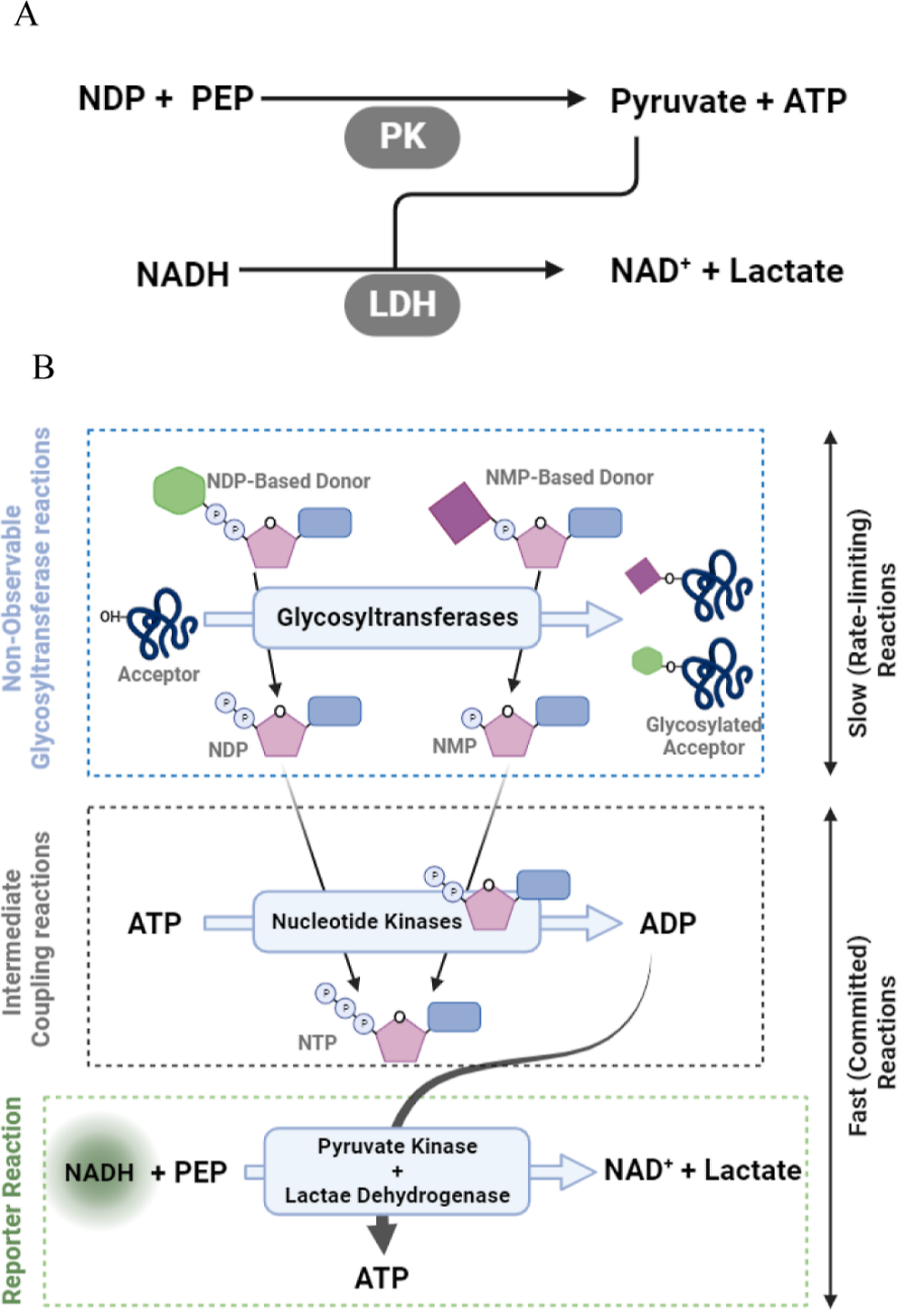
Principle of the UGC assay. (A) PK/LDH coupling reaction. (B) The nonobservable glycosylation reaction is detected in real-time via coupling the released nucleotides to the reporter coupled reaction PK/LDH. The coupling is achieved via an intermediate nucleotide kinase that phosphorylates the nucleotide and produces ADP.

The coupled assay was constructed and optimized by following a systematic bottom-up approach. The detection mode of NADH was shifted from absorbance to fluorescence. Computational modeling of PK/LDH coupling was performed to establish the parameters of this coupling step, which was validated experimentally. The coupling of each of the nucleotides, released as products from every GT class, to PK/LDH was redesigned for optimal performance. This was achieved by employing cytidylate kinase (CMK), a nucleoside kinase, and the nucleoside diphosphate kinase (NDK) in nucleoside-base-determined combinatorial modules that delivered an optimized coupling for each GT class. The resultant assay design presented here is the first UGC coupled assay that can be easily deployed as a standardized tool for studying GT activity. In addition, the UGC assay can be adapted into a high-throughput format for drug screening after optimizing a robust signal range and controlling for possible nonspecific inhibition at the intermediate and/or the reporter reaction levels of the coupling assay (Figure 2).

## 2 Results and Discussion

The GTs facilitate the transfer of monosaccharides from an activated nucleotide donor to a glycosyl acceptor molecule, resulting in the production of a free nucleotide and a glycoconjugate. These reactions, known as Bi–Bi reactions, involve two substrates and two products. While the acceptors display diversity, the donors can be categorized as either nucleoside diphosphate (NDP)-based, in the form of a UDP/GDP-based donor, or nucleoside monophosphate (NMP)-based, in the form of a CMP-based donor. CMP-based reactions are specific to STs, GDP-based reactions are specific to fucosyltransferases and mannosyltransferases, and UDP-based reactions encompass the remaining GT reactions.^9^

The main challenge in developing assays for GT enzymes is that none of the substrates (donor and acceptor) or the products have a differentiating optical property, fluorescence, or absorbance. Several assays have been developed to investigate the kinetics of GTs, but they suffer from drawbacks such as being end point assays or continuous assays with compromised specificity, sensitivity, or cost.^10−12^ A Transcreener end point assay for immunodetection of UDP with a time-resolved Förster resonance energy transfer signal that measures UDP via antibodies selective to UDP produced by GT was developed. This commercial assay has been developed for high-throughput screening discovery of GT inhibitors.^13^ Other popular commercial assays are UDP-Glo, and its recently released versions GDP-Glo and UMP/CMP-Glo GT assays provide a method to rapidly and sensitively detect UDP, GDP, and CMP formation in GT reactions.^14^ The objective was to profile GT specificity for different sugars, screening library compounds for effects on GT activity, and detecting chemical compound glucuronidation during drug discovery. The current assays have limitations and are not applicable for standardized universal GT high-throughput screening and big data gathering. However, some are very well suited for GT high-throughput screening when accurate end point readouts are the only objective. Specifically, Promega’s UDP-Glo, GDP-Glo, and UMP/CMP-Glo are optimized for this purpose. But when the objectives are to undertake kinetics measurements or to document dynamic drug–enzyme interactions, these end point assays are suboptimal. To study the kinetics of the ST, ST3GAL1, Ortiz-Soto et al. (2019)^15^ modified a continuous enzyme-coupled assay that was originally developed by Gosselin et al. (1994)^16^ and optimized by Brown et al. (2012)^17^ as a generic nucleotide-based assay. This assay is based on coupling the nucleotide released from the GT reaction with pyruvate kinase (PK) directly in the case of UDP/GDP-based donor reactions. In the case of CMP-based reactions, the coupling is carried out via another kinase, nucleoside monophosphate kinase (NMPK), which phosphorylates CMP to CDP in the presence of excess ATP. The resulting influx of NDP (UDP, GDP or ADP) to PK reaction converts phosphoenolpyruvate (PEP) to pyruvate, which is used to oxidize NADH by lactate dehydrogenase (LDH) (Figure 2A).

### 2.1 Assay Development and Optimization

The principle of an enzymatic-coupled assay is that a nonobservable reaction of interest is coupled with multiple intermediate enzymatic reactions where the product(s) of one reaction serve(s) as a substrate for the subsequent reaction. The chain of reactions ends with a reporter reaction that involves one detectable species that can be accurately measured either in real-time (continuous assay) or after developing a signal, which requires termination of the reaction (end point/noncontinuous assay).

The main condition needed to validate continuous enzymatic-coupled assays is that the steady state of the system is maintained. That is the reaction of interest (the nonobservable reaction) must be the rate-limiting step and the subsequent reactions (intermediate and reporter reactions) must be sensitive and fast enough (committed) that intermediate products are instantly and spectroscopically detectable. The smaller amount of the latter is therefore a measure of the assay’s sensitivity. To achieve these conditions, the enzymes in the subsequent reactions must be chosen to have low *K*_m_ and high *k*_cat_ values for their substrates (the intermediate products) in the enzyme chain. To achieve universality, these criteria must hold for a defined and broad range of the nonobservable reaction rates delivering accurate measures of kinetics parameters for every target reaction.

The adherence to the above validation criteria was investigated for assays that lead up^15,16,18^ to our current UGC assay development. In these legacy assays, GT/PK/LDH and GT/CMK (or NMPK)/PK/LDH coupling formats were used for reporting UDP/GDP-based donor and CMP-based donor GT reaction classes, respectively. It was concluded that previous designs suffer from lags and/or insensitive coupling of the nucleotides to PK/LDH. Consequently, the design used in the UGC assay relies on complete phosphorylation of the nucleotides to nucleoside triphosphate (NTP), which takes place in a single step in the case of the NDPs and in two steps in the case of CMP (Figure 2B). In addition to the novel adaptations to the design, the assay was optimized through a systematic bottom-up approach that included NADH detection and PK/LDH coupling parametrization.

#### 2.1.1 NADH Signal Optimization

The concentration of NADH, as the reporter molecule in the coupling assay, is conventionally determined by measuring the absorbance at 340 nm. The absorbance method for quantifying NADH in PK/LDH coupled assays has limitations in both sensitivity and specificity. In miniaturized assays, such as the 384-well plate format, the short optical path length diminishes absorbance signal strength, compromising assay sensitivity.^19^ Moreover, absorbance is susceptible to interference from molecules with overlapping spectra, including sample particulates that cause light scattering, resulting in a poor signal-to-background ratio. This interference is especially problematic in drug screening due to challenges in identifying and controlling for it across all screened molecules,^19,20^ The NADH’s intrinsic fluorescence^21^ allows for these methods of detection to overcome the many limitations of absorbance.^22^

In this work, a fluorescence protocol was optimized for NADH quantification by measuring fluorescence at excitation and emission wavelengths of 340/460 nm. The sensitivity of the assay enabled miniaturizing the reaction volume to 30 μL in a 384-well plate. Fluorescence linearity in correlation to concentration was found to hold to a concentration of NADH up to 150 μM, with ideal linearity below 100 μM (see Supporting Information Figure S1B). In addition to linearity, the initial concentration of NADH must be maximized to sustain a rapid turnover of the pyruvate generated by PK reaction via LDH. While manufacturer kinetics parameters of NADH were not reported, the curated values of *K*_m_ on the Brenda database show a median of 58 μM (see Supporting Information Table S1). Consequently, the depletion of NADH in a range of around 100 μM is expected to yield a relatively stable turnover rate, especially when LDH is provided in abundance. The stability of the NADH signal was confirmed for an incubation period of 30 min (Figure S1A, Supporting Information).

#### 2.1.2 PK/LDH Coupling Optimization

The aim of this optimization step is to ensure that the PK/LDH coupled reaction shown in Figure 2B as the reporter reaction in the assay is, indeed, a committed step. To achieve this the sensitivity of the PK/LDH coupled reaction was maximized to detect and report ADP in real-time as well as maintaining linear performance for a defined range of ADP concentrations. This is done by supplying the substrates and enzymes in abundance. Which is applicable only for the substrates PEP and ATP but not for the NADH whose concentration is limited by the linearity of its fluorescence (shown in Section 2.1.1). To limit wasteful use of PK and LDH and to minimize the reaction crowding effect^23^ their relative concentrations were titrated until the optimal ratio was reached. In addition, limiting the use of PK and LDH minimizes the introduction of unnecessary buffer components and impurities included in the enzyme stock solutions.

##### 2.1.2.1 Optimizing of PK/LDH Coupling Parameters from Computational Modeling

To explore the effect of NADH concentration on the assay performance, a simulation model of the coupled assay in batch mode was designed and performed using the COPASI systems chemical biology package.^24^ Initial concentrations were set as follows: one unit of each of PK and LDH (1:1 ratio), 2 mM of PEP, 2 mM of ATP, and varying concentrations for NADH and ADP. The definition of one unit of PK and LDH is detailed in the Method Section. Initial concentrations of pyruvate, lactate, and NAD^+^ were set to zero. ATP was added to account for the nucleotide kinase coupling reactions (Figure 2) since ATP is a product of PK reverse reaction, its inhibitory effect on the forward reaction must be factored in the modeling.

**Figure 3:**
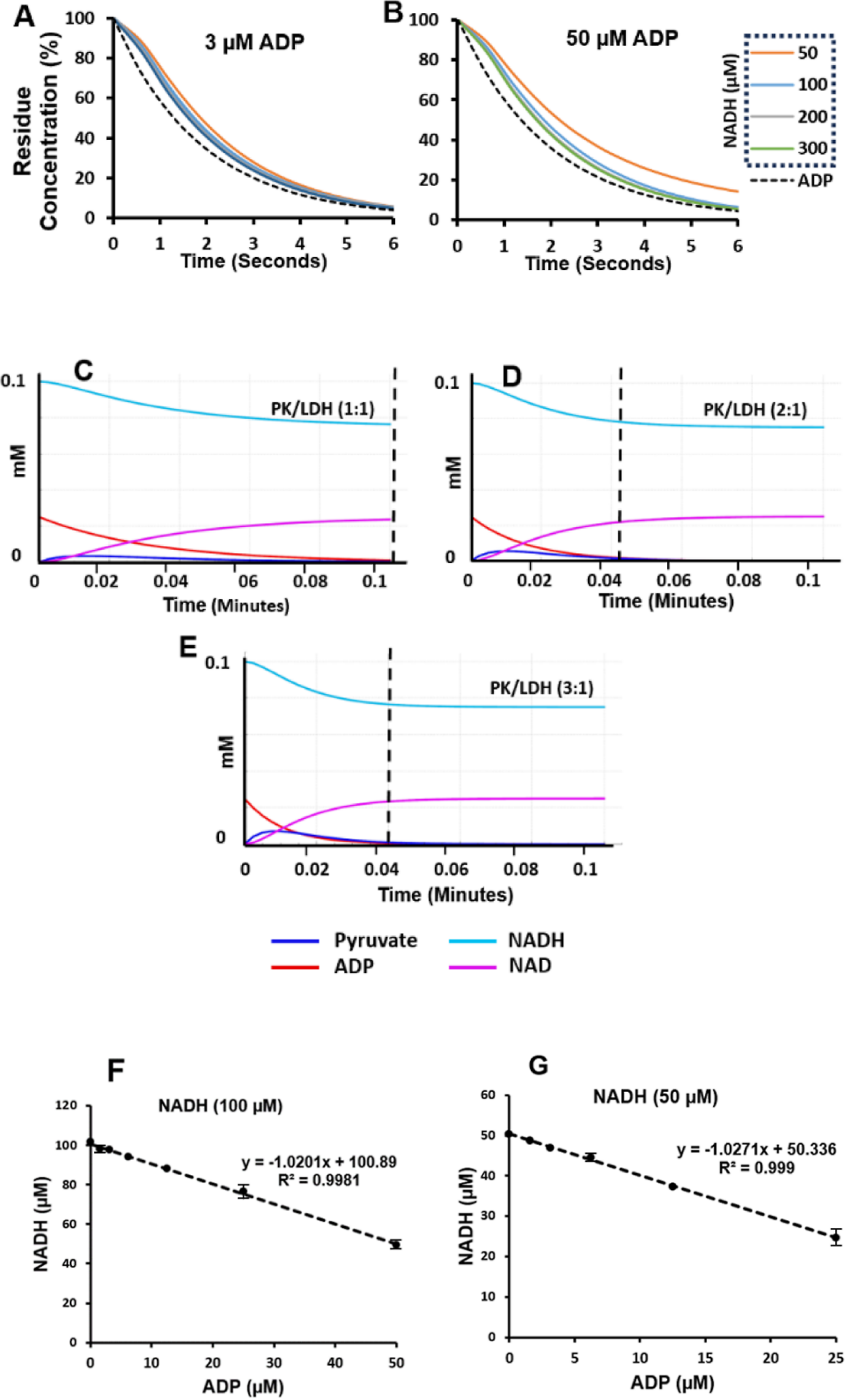
Optimization of PK/LDH coupling. (A) NADH concentration effect on PK/LDH performance in depleting ADP at two initial concentrations: 3 μM and (B) 50 μM. Solid lines are the depletion of NADH. Dashed line is the depletion of ADP (chosen for 100 μM initial concentration of NADH as ADP depletion was NADH-independent). Concentrations on the *Y* axis are presented as a percentage of the residue concentration (explained in the Methods). (C–E) The effect of PK/LDH ratio on the ADP depletion lag time. The vertical dashed lines indicate full depletion of ADP. (F,G) Linear regression of ADP vs NADH concentration determined experimentally when 100 or 50 μM NADH was used, respectively. The data in panels F and G are presented as means ± SD, *n* = 3.

Both ATP and PEP were supplied at near saturating concentrations, being more than 20-fold of the *K*_m_ values for the nucleotide kinases (NDK and CMK), and the PK used in this study, respectively (Supporting Information Table S1). These conditions were used to simulate the response of the system, represented by NADH depletion, coupled with the addition of either 3 or 50 μM ADP. The simulation was repeated for four concentrations of NADH: 50, 100, 200, and 300 μM (Figure 3A,B). The results show that LDH was very responsive to PK flux, as indicated by the small elevation of pyruvate during the course of the reaction (Figure 3C) and the small lag between the NADH depletion curves and the ADP depletion. The results also suggest that the activity of LDH was not affected by NADH concentrations in the tested conditions. However, using higher concentrations of ADP (e.g., 50 μM) then decreasing the NADH concentration (to 50 μM) led to a significant increase in lag time of NADH depletion. This is due to the linear substrate-dependency of the enzymatic reaction rate at substrate concentrations below its *K*_m_ (see Figure 3B and Supporting Information Table S1). None the less, despite the responsiveness of NADH to ADP, there remained a significant overall lag in the system of more than 6 s. An even longer lag time may be observed in experiments since factors such as substrate diffusion, suboptimal activities of the enzymes, and the individual time for each enzyme to reach its own steady state are not included in the simulation model.

Further testing the assumption that PK is the rate-limiting step, the objective was then to reduce this lag time using the finding of this exploratory simulation. The time course simulation was then repeated for 25 μM ADP and 100 μM NADH (i.e., the average concentrations used in the exploratory simulation). Conditions for PEP, ATP, pyruvate, and NAD^+^ were kept unchanged from the previous test. LDH was fixed at 1 unit with PK tested at three concentrations: 1, 2, and 3 units. The results (Figure 3C–E) show that increasing the ratio of PK to LDH from 1:1 to 2:1 produces a significantly reduced lag time. Reductions in lag time of 2.5 s for 2:1 and more than 6 s for a ratio of 1:1 were observed, but no further improvement was detected for a 3:1 ratio (Figure 3D,E). Contrary to most reported protocols using higher LDH to PK concentrations the conclusions of these simulations imply that PK to LDH should be used in a 2:1 ratio.

##### 2.1.2.2 Experimental Validation of PK/LDH Coupling Parameters

The performance of the reaction was tested with a 2-fold serial dilution of ADP at different NADH concentrations using the 2:1 ratio of PK to LDH (6:3 units) (see Supporting Information Figure S2). The signal progress cannot be recorded instantly after initiation of the reaction because of limitation in the experimental setup. Consequently, a delay of ∼10 s was practically imposed. The lag time frame observed in the simulation could not be experimentally duplicated. Given this experimental time delay, all combinations the reactions were then observed to be complete or at near complete by the time the signal recording started. However, the lag in ADP/NADH depletion with 50 μM NADH when ADP was used at high concentrations, observed in the simulation, was confirmed experimentally.

All progress curves (Supporting Information, Figure S2) plateaued at stable fluorescence signals. In addition, the signals showed a linear correlation with the concentrations of ADP except when ADP concentration approaches that of NADH in the mixture (Supporting Information Figure S2B). For ease of comparison, the fluorescence is converted to the NADH concentration using the NADH standard curve (Supporting Information Figure S1). The linear regression of ADP vs NADH revealed a 1:1 depletion of NADH to ADP that is independent of NADH concentration. This is illustrated for 100 (Figure 3F) and 50 μM (Figure 3G).

### 2.2 Coupling of UDP and GDP

In addition to ADP as its main substrate, PK can phosphorylate other NDPs, such as CDP, UDP and GDP. This reactivity of PK was the principle of the original design proposed by Grosselin et al. 1994.^16^ However, the efficiency of the phosphorylation of GDP and UDP by PK in comparison to that of ADP has not been thoroughly investigated. The relative value of *K*_m_ for GDP to ADP is reported to be higher in some publications^25,26^ and in the same range (as medians) for the values curated in the Brenda database (Table S1 in Supporting Information). The data on the Brenda database suggests low affinity to UDP. In one study^27^ GDP and UDP were phosphorylated by another enzyme, NDP kinase from baker’s yeast. This phosphorylation is done by ATP, and it releases ADP in an equivalent amount to GDP/UDP.

Here we first confirm the limitation of the direct coupling of UDP and GDP to PK/LDH. Following this, we explore the use of another kinase, NDK, which was used to phosphorylate GDP and UDP to GTP and UTP, releasing ADP in the process. A survey of the Brenda database of kinetics parameters for all NDK enzymes NDK (EC 2.7.4.6) was conducted to find the isozyme that meets the performance criteria: (1) having a low *K*_m_ value for UDP/GDP that is less than that of PK guaranteeing that the NDK reaction is the committed step, (2) having a higher *K*_m_ value for ADP than that of PK so avoiding capturing ADP preventing its access to PK, (3) having a low *K*_m_ for ATP, (4) having a high *K*_m_ for UTP/GTP to minimize the possibility of phosphorylation of ADP with UTP/GTP and finally (5) being easily expressed in *Escherichia coli* i.e., lacking eukaryotic post-translational modifications requirement. Specifically, the survey led to the selection of *Acanthamoeba polyphaga Mimivirus* ortholog (*K*_m_ values shown in Table S1 Supporting Information). The *Mimivirus* NDK has a *K*_m_ value for UDP 10-fold lower than that of PK and 3–4 times higher for ADP than that of PK. It also has a very low *K*_m_ for ATP and relatively high *K*_m_ for UTP and GTP with values of 0.1 and 0.83 mM, respectively.

**Figure 4:**
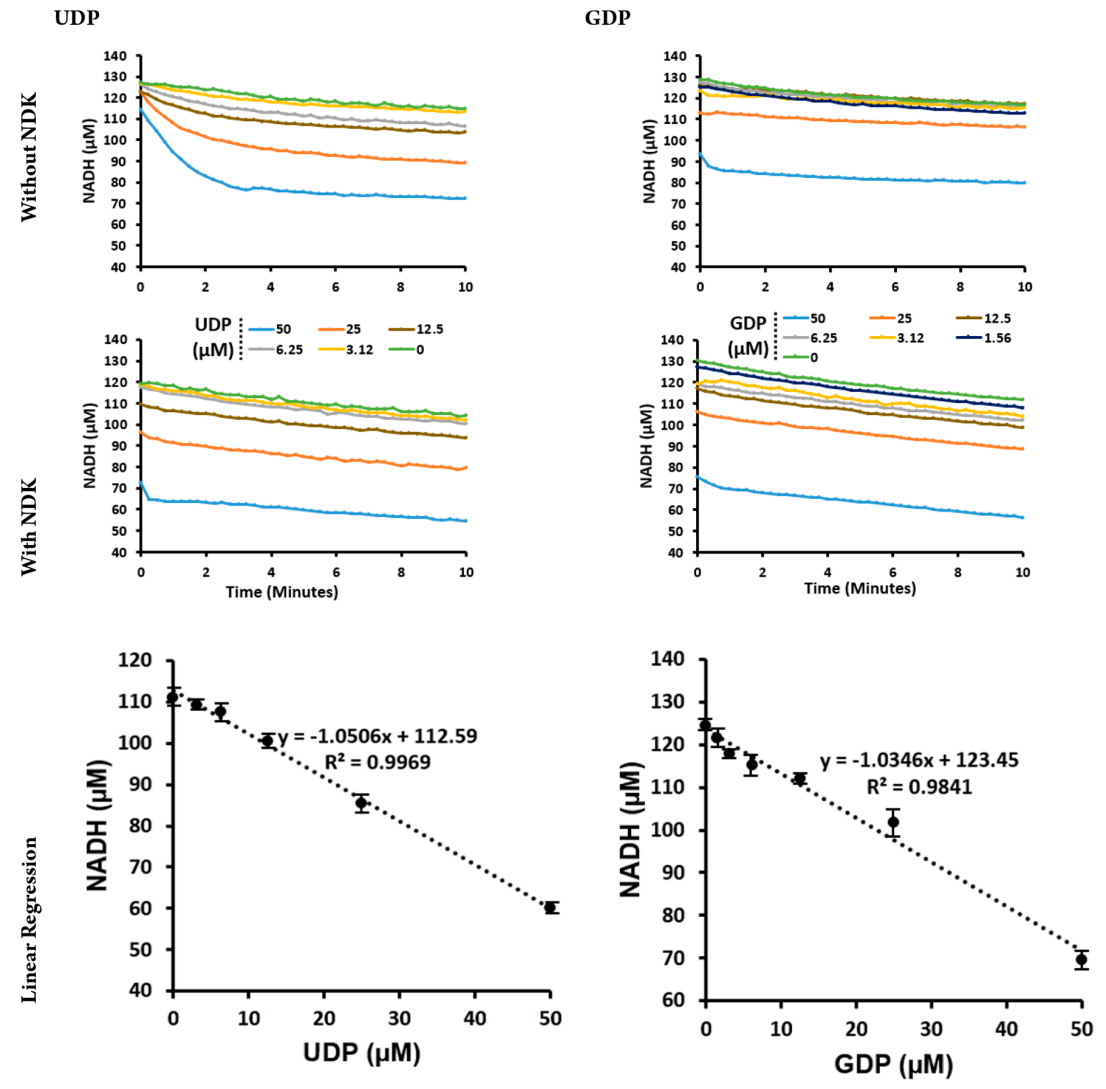
Time course of the PK/LDH reaction with a 2-fold serial dilution of UDP (left column) and then GDP (right column) performed without and then with NDK (500 nM). Linear regression of NADH vs ADP/UDP and NADH vs ADP/GDP concentration in the presence of NDK is shown in the bottom panels. Fluorescence signal averaged over 10 min were converted to NADH concentrations using the slope given in Figure S1 (Supporting Information). The linear regression data in panels are presented as means ± SD, *n* = 3.

To investigate the role of NDK in UDP and GDP phosphorylation, serial dilutions of both nucleotides were reacted with reaction mixtures in the presence or absence of 500 nM NDK (Figure 4). The effect of NDK in eliminating the lag time was confirmed for the range of UDP tested concentrations. In the case of GDP, the addition of NDK increased the sensitivity of coupled reaction to GDP. NADH depletion showed a linear relation to the concentration of UDP and GDP added to the reaction with a 1:1 ratio, seen from the slope of the regression (Figure 4). The addition of NDK caused a background NADH depletion, which is independent of the NDP as can be seen from the parallel plots in each set. This effect is a slow NDK-dependent (Figure S3) effect possibly due to an NDK stock- and buffer-dependent reaction related to an unknown impurity in the stocks. None the less, the resultant background depletion rate remained stable during the course of signal recording (Figures S3 and S4) and has no impact on the validity of the assay as confirmed by the linearity of NADH vs UDP/GDP. The important takeaway is that the activity of PK by itself is insufficient to provide rapid and sensitive detection of UDP/GDP. Integrating NDK provides an efficient route that is faster and more sensitive at achieving UDP/GDP phosphorylation than does direct phosphorylation with only PK.

### 2.3 Coupling of CMP

The coupling of CMP to the PK/LDH reaction has previously been accomplished using the NMPK enzyme from the baker’s yeast (EC 2.7.4.4).^15,16^ This is a problematic workflow since the baker’s yeast NMPK is not easily commercially sourced and does not have a complete kinetics parameters profile. Further, this previously used coupling step was not characterized or optimized. We found that cytosine kinase (CMK: EC 2.7.4.25) from *E. coli* to be a superior alternative with low *K*_m_ values for ATP and CMP (Table S1, Supporting Information). It has a comparatively greater chance of successful expression in an *E. coli* expression host. Moreover, CMK’s *K*_m_ value for CMP is much lower than for other nucleotides, making it highly specific and thus minimizing the chances of unwanted side reactions.

**Figure 5:**
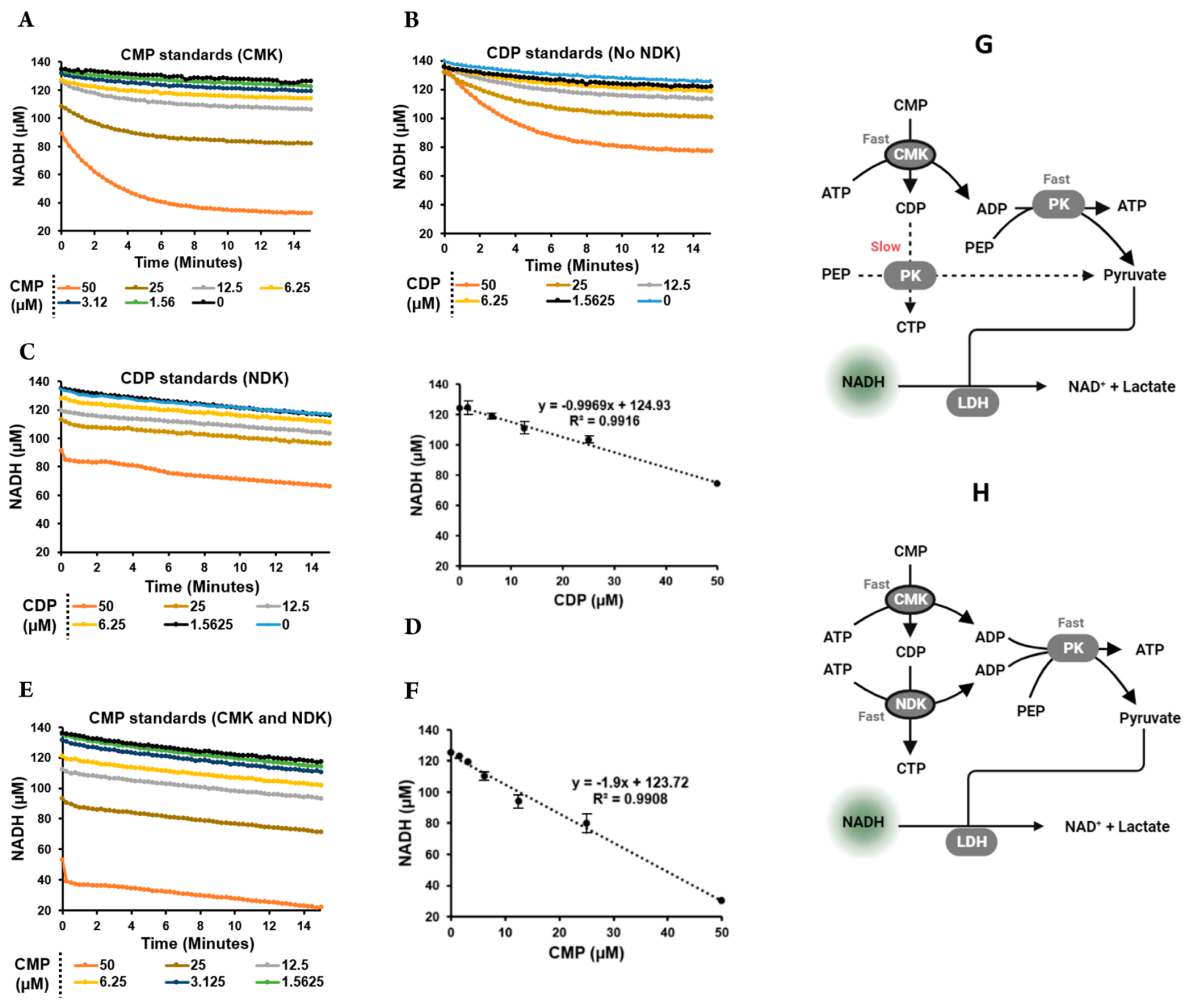
Development of CMP coupling assay. (A–E) Progress curves of the response of the different enzymatic-coupled format to the different substrates: CMK/PK/LDH to CMP, PK/LDH to CDP, NDK/PK/LDH to CDP, and CMK/NDK/PK/LDH to CMP, respectively. (D,F) Show the linear regression of the averaged NADH concentrations over 10 min vs the concentration of the substrates corresponding to panels (C,E) respectively. (G,H) Are the schematic representations of CMP coupling via CMK/PK/LDH and via CMK/NDK/PK/LDH, respectively. The data in panels D and F are presented as means ± SD, *n* = 3.

CMK was coupled to PK/LDH by adding CMK to the PK/LDH optimized mixture. The response of CMK/PK/LDH to a serial dilution of CMP was tested (Figure 5A). The progress curves show initial depletion of NADH corresponds to the CMP concentration (1:1 ratio), followed by further slow depletion up to 6 min. The depletion is completed when the final consumption of NADH corresponds to a 2:1 ratio of added CMP. This observation was made by monitoring two reactions. First, the initial instant phosphorylation of CMP to CDP via CMK followed by a slower phosphorylation of CDP via PK (Figure 5G). This was confirmed through the response of the PK/LDH reaction mixture to a serial dilution of CDP (Figure 5B). The same lag time observed in panel A was also observed with CDP progress curves, confirming the predicted slow CDP phosphorylation by PK. An alternative route of CDP phosphorylation by NDK was explored, where the NDK was added to a final concentration of 500 nM to the PK/LDH reaction mixture, and the response to CDP was repeated as before (Figure 5C). In this exploratory experiment, the depletion of NADH was instant and the linear regression (Figure 5D) showed a depletion of NADH corresponding to a 1:1 ratio of CDP. Confirming the observed efficiency, the response of the CMK/NDK/PK/LDH coupled assay to a serial dilution of CMP was performed (Figure 5E).

The progress curves showed that the instant linear depletion of NADH corresponds to a ratio of approximately 2:1 of the CMP concentration (Figure 5F). The addition of NDK resolved the problem of the secondary slow phosphorylation of CDP in the CMK/PK/LDH format by bypassing the slow reaction and replacing it with the committed fast phosphorylation of CDP by NDK (Figure 5G,H). This result proves the CMK/PK/LDH format as a suboptimal coupled assay for CMP detection in real-time in comparison to the case when NDK is inserted in the workflow. The CMP coupling component of the assay has therefore been fully optimized.

### 2.4 UGC Coupling Assay

**Figure 6:**
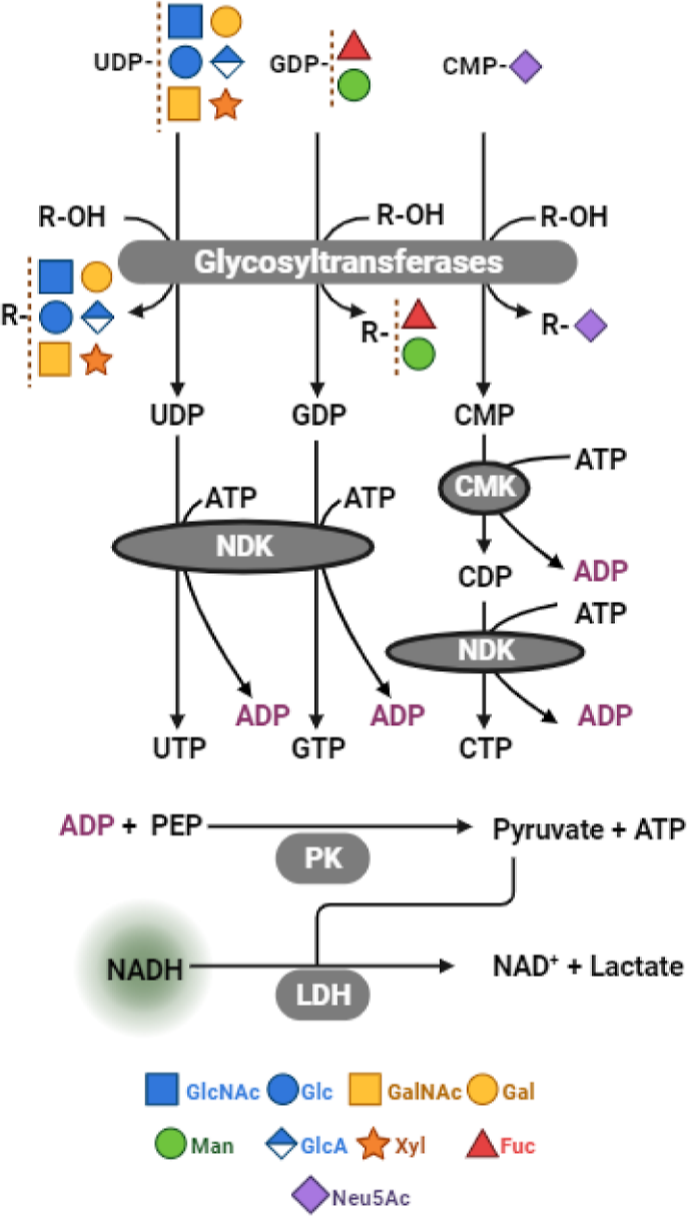
Schematic representation of the UGC coupled assay. Reading from top-down GTs catalyze the transfer of a monosaccharide moiety from an activated sugar nucleotide to an acceptor, releasing UDP, GDP, or CMP. In the next coupling step, the released nucleotides are phosphorylated to their corresponding NTPs UTP, GTP, or CTP respectively, via nucleoside diphosphate kinase (NDK) and cytoidylate kinase (CMK) that phosphorylate released nucleotides with ATP to release ADP. The final coupling step in the assay employs LDH enzymes PK and LDH that couple released ADP to NADH depletion. The drop in the fluorescence signal of NADH is monitored.

We now conclude that the enzymatic coupling to measure the reactivity of all classes of GTs catalyzing glycosylation reactions from sugar donors bound to UDP, GDP, and CMP has been rigorously optimized. This UGC assay can be used to accurately and with significant sensitivity measure glycosylation as well as the inhibition thereof. The design of the coupling assay is summarized in Figure 6.

#### 2.4.1 Application of the UGC Assay in ST3GAL1, C1GALT1, and FUT1 Kinetics Measurements

The GT enzyme reactions undergo Bi–Bi mechanisms (i.e., two substrates-two products). The kinetics parameters for these reactions are therefore investigated by supplying in turn saturating concentrations (5–10 times its *K*_m_) of one substrate at a time while varying the concentration of the other substrate. When the *K*_m_ values of a substrate are not known then iterative parameter and saturating concentration adjustments are needed to reach validation where the *k*_cat_ values measure from each of the substrates converge relatively close to each other.

**Figure 7:**
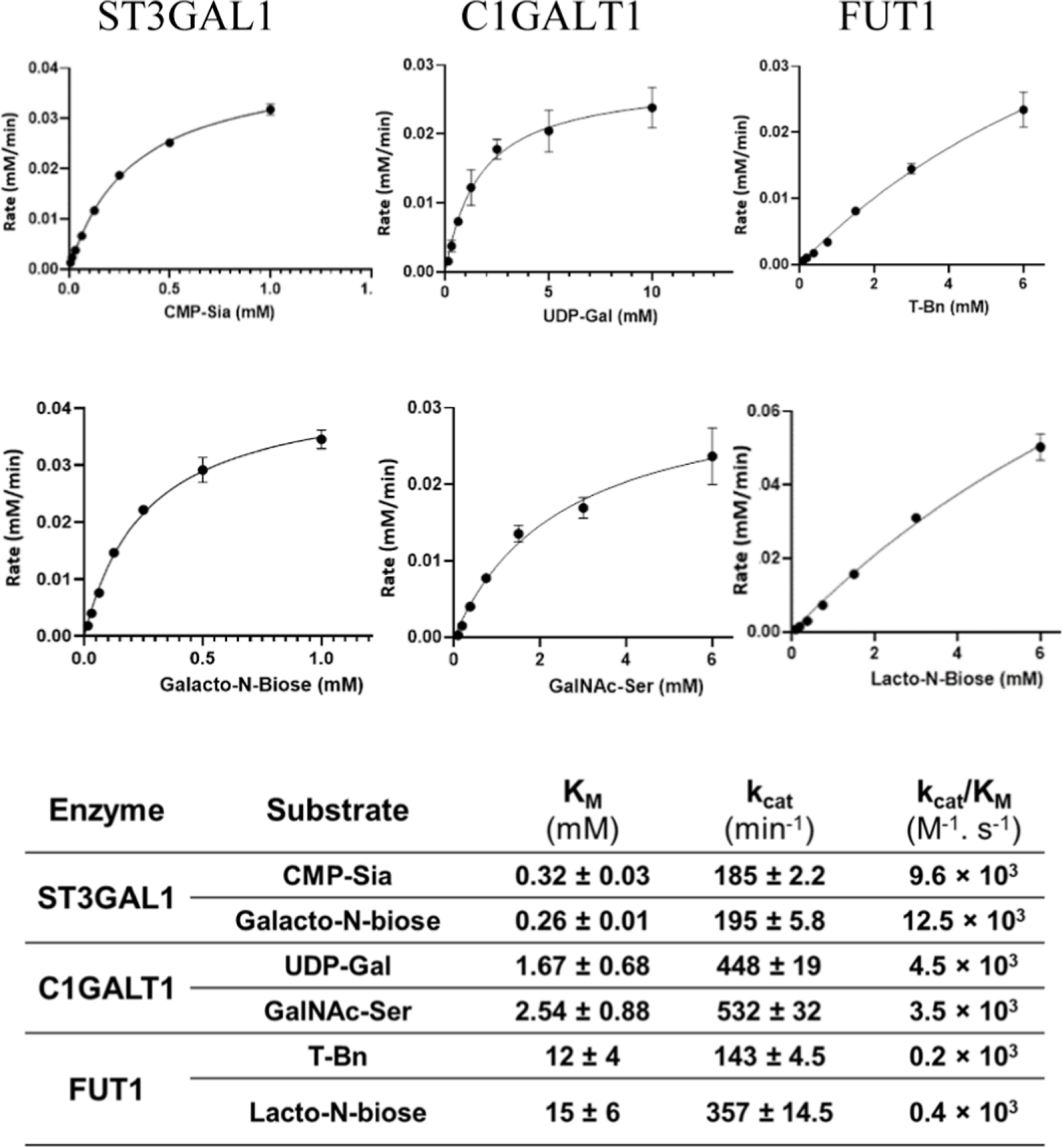
Direct plotting of reaction rates vs substrate concentration for all of the enzyme–substrate profiles tested in this study. Concentration of the substrates is explained in the Method Section. The rates were calculated from converting the rate of NADH oxidation (depletion) to the rate of the released nucleotide (Figure S4, Supporting Information). The kinetics parameters were calculated by fitting the data to the Michaelis–Menten model (Figure S5, Supporting Information). The table in the figure is a summary of the calculated parameters. The data is presented as means ± SD, *n* = 3. Distribution normality was tested using the Shapiro–Wilk test.

The efficacy of the UGC assay is applied to the kinetic study of a system of three GTs: ST3GAL1, C1GALT1, and FUT1. These represent GT sugar nucleotide donor classes CMP, UDP, and GDP, respectively. Both C1GALT1 and ST3GAL1 (also known as core 1 synthase) are involved in the Mucin-Type *O*-glycosylation pathway. This pathway is deregulated in many cancer types, such as breast and colon cancers. Their products, Core1 (T antigen) and sialyl T antigen (ST) have been well established as diagnostic and prognostic markers. Consequently, their roles in carcinogenesis have been the subject of numerous studies.^28^ Here the kinetics for the three enzymes were measured by using the UGC assay in its NDK/PK/LDH format for C1GALT1 and FUT1 and in its CMK/NDK/PK/LDH format for ST3GAL1. The assay was performed as described in the methods section and is illustrated on ST3GAL1 with detailed reporting given in (Figures S4 and S5, Supporting Information). Direct plots of the rates versus substrate concentrations and summaries of the kinetics parameters for all three enzyme–substrate pairs are shown in Figure 7.

The ST ST3GAL1 transfers sialic acid (Neu5Ac or Sia) from the CMP-Sia donor to Galβ1-3GalNAc or Galβ1-3GalNAc-*O*-Ser/Thr acceptor (T antigen).^29^ In this study, the substrate saturating concentrations for both the acceptor and donor in the case of ST3GAL1 (I mM for both) were approximately 4 and 3 times the calculated *K*_m_ values, respectively. Despite being suboptimal, these saturating concentrations of *k*_cat_ values for the donor CMP-Sia (*k*_cat_ = 185 min^–1^) and acceptor Galacto-*N*-Biose (*k*_cat_ = 195 min^–1^) reach near convergence (Figure 7). These are close to the values reported previously for ST3GAL1 expressed in *E. coli*.^15,30^

C1GALT1 transfers galactose from a UDP-Gal donor to the GalNAc-Ser/Thr (Tn antigen) acceptor. The kinetics of these substrates for human C1GALT1 are not known. To measure these parameters for C1GALT, the acceptor’s saturation concentration was assumed to be around the value for *K*_m_ which led to an underestimation of the *k*_cat_ for the donor. However, in the paired reaction rate measurement when using 6 times the donor *K*_m_ value as the saturating concentration, a higher *k*_cat_ value was produced for the acceptor. The cost and availability of substrate prevented sufficient acceptor’s saturation concentration conditions being reached. Consequently, the parameters calculated from sub saturating conditions are recorded here as apparent parameters.

FUT1 is a galactoside α-(1,2)-fucosyltransferase that fucosylates galactose in a variety of acceptor substrates, such as Galβ1-3GlcNac, Galβ1-4GlcNac, and Galβ1-3GalNAc.^31^ FUT1 expression is altered in many cancer types and its altered regulation has been correlated to cancer progression.^32^ Kinetics parameters for FUT1 were calculated with a saturation concentration of the donor GDP-Fuc and variable concentrations of the acceptors Galβ1-3GalNAc-*O*-benzyl (T-Bn) and Galβ1-3GlcNac (lacto-*N*-biose). Saturating concentrations for both acceptors were limited by material availability. This resulted in an uncertainty in the computation of *K*_m_ values from Michaelis–Menten fittings (Table S2, Supporting Information). None the less, the best fit values calculated using this, albeit limited data set, shows a slightly lower *K*_m_ for T-Bn compared with lacto-*N*-biose and agrees with the trend reported by Sarnesto et al., 1992.^31^

Apart from its role in cancer progression, the product of FUT1 glycosylation on the surface of red blood cells (antigen H), is the precursor of the blood groups A, B, AB, and O. A comparison of reactivity of the two acceptors in FUT1 (Figure 7) shows lacto-*N*-biose producing significantly higher reaction rates (*k*_cat_ = 357 min^–1^ cf. *k*_cat_ = 152 min^–1^ for T-Bn) and a greater catalytic efficiency (*k*_cat_/*K*_m_ = 0.4 M^–1^ s^–1^ cf. *k*_cat_/*K*_m_ = 0.2 M^–1^ s^–^1 for T-Bn). This preference to Galβ1-3GlcNac (type 1H antigen), over Galβ1-3GalNAc was previously reported,^33^ and concurs with the consensus view that the synthesis of the antigen H is the primary role of FUT1.

A significant improvement in accuracy measuring initial rates by continuous assays over end point (noncontinuous) assays is a major advantage for the former method. Using end point assays to determine accurate rates is an extremely challenging task, especially in the case of fast reactions. A case in point is calculating rates at the higher end of the substrate concentration range using the direct blotting method. End point assays are prone to imprecisely identifying the linear range of the reaction progress curve, which results in an underestimation of the real initial rate. This methodological weakness leads to direct blotting curves with an apparent early plateauing of rates at lower concentrations. What follows is a lower estimation of *K*_m_, *V*_max_, and *k*_cat_ values. Currently, the overwhelming majority, if not all, of the kinetics parameters reported for GTs are determined using end point assays. By way of example in this work, the *K*_m_ values for FUT1 (Figure 7) determined from the UGC assay are significantly higher by comparison to that from an end point assay reported by Sarnesto et al., in their 1992 study.^31^

The uniformed detection mechanism underlying the design of the assay (Figure 6) demonstrate an important feature of the UGC assay to generate standardized kinetics parameters making possible inter/intra pathway and intersubstrate comparative and mechanistic studies.

#### 2.4.2 Application of the UGC Assay in Inhibition Measurement of Soyasaponin 1 on ST3GAL1, C1GALT1, and FUT1

GTs are a large family of enzymes sharing structural and functional similarities,^34^ this makes the discovery of GT specific inhibitors complicated. An obstacle to discovering GT specific inhibition is the absence of a standardized assay, where kinetics measurements can be made on one experimental platform. Such an assay allows for direct comparison of the small molecule binding and reaction inhibition between GTs. To demonstrate this, the efficacy of the UGC assay as a standardized inhibition assay for GTs is applied to the natural product soyasaponin1 inhibitor model that was previously reported as being an effective and selective inhibitor of ST3Gal1.^3^ Soyasaponin1 was discovered from an in vitro high-throughput screening campaign of natural products for their inhibitory effect on STs.^35^ In the study, Wu et al.^35^ determined the ST specificity of soyasaponin by performing a comprehensive dose response study using an end point assay approach against different STs as well as examples of fucosyltransferases and galactosyltransferases. The rationale for choosing the reaction incubation time and the concentrations of the substrates used in the end point assay performed in the study was not explained.

**Figure 8:**
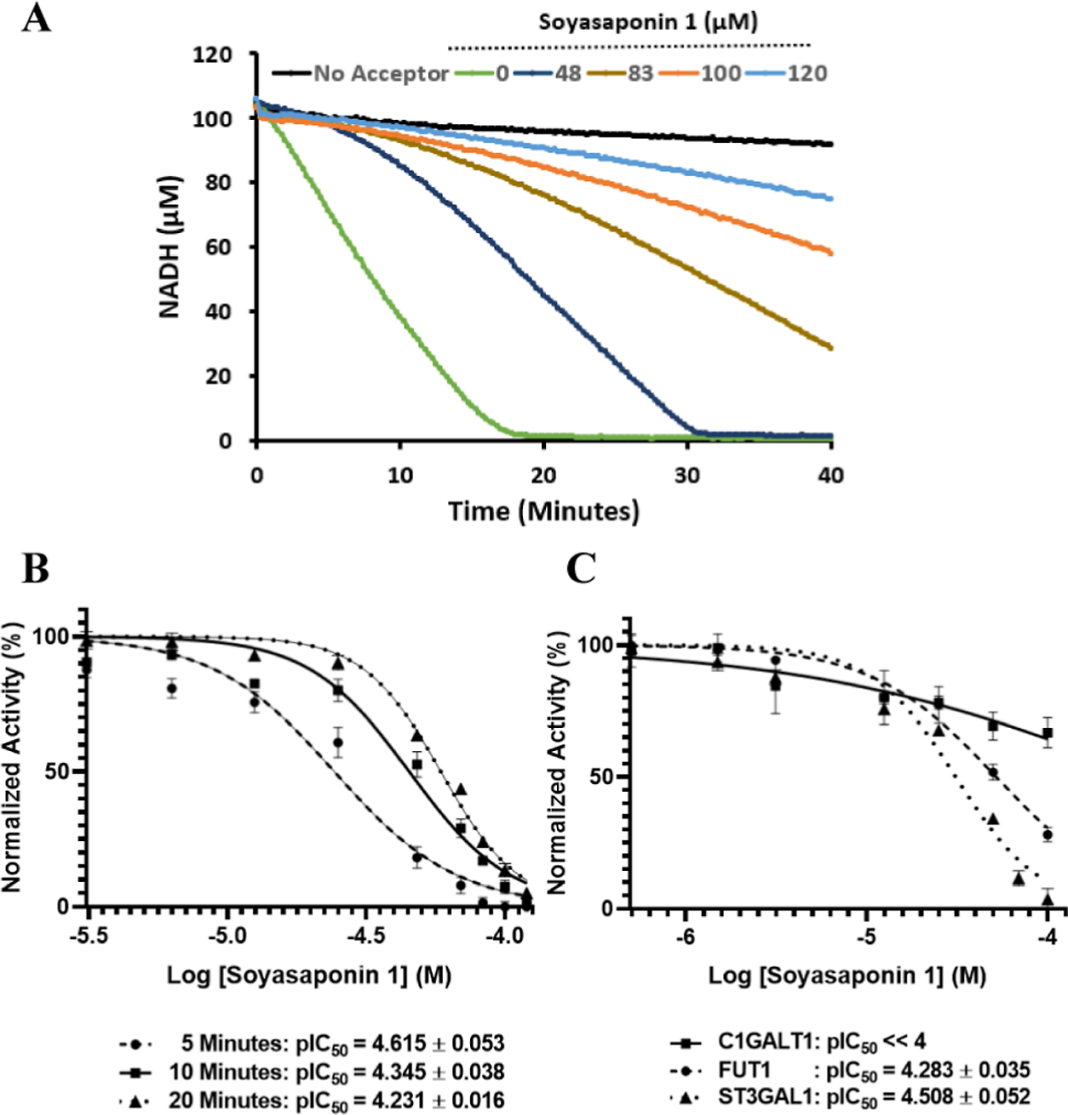
Soyasaponin1 inhibitory effect on ST3GAL1, C1GALT1 and FUT1. (A) Progress curves showing the dose and time dependency of Soyasaponin1 inhibition on ST3GAL1 enzymatic reaction with concentrations of the substrates equal to their *K*_m_ values. A control reaction without an acceptor was included. (B) The dependency of dose response (and pIC_50_) on incubation time. Details of the fitting and calculations are in the Methods and Table S3 (Supporting Information). (C) Dose response of ST3GAL1, C1GALT1, and FUT1. Concentrations in panels B and C are in log scale. The data in panels B and C are presented as means ± SD, *n* = 3 except for the pIC_50_ values that are presented as means ± SEM.

This is a significant shortcoming in the study, which we uncovered from the dose response of ST3GAL1 to the serial dilution of Soyasaponin1. First, when Soyasaponin1 and the substrates were simultaneously added to the enzyme, no inhibitory effect was observed within its solubility range. As a result, a procedure consistent with the original study^35^ was used where Soyasaponin1 was preincubated with the enzyme prior to initiating the reaction with the substrates added to the detection mixture. Time and dose dependent inhibition was measured at varying concentrations of Soyasaponin 1. The reaction mixtures contained both the acceptor and donor at concentrations equal to their *K*_m_ values (Figure 8A). However, when the dose response is calculated at different time points, the IC_50_ values show a clear time dependency (Figure 8B); the IC_50_ values are higher as the incubation time increases.

The preincubation is a necessary condition for producing the inhibitory effect on ST3GAL1, which can be indicative of a slow binding of the Soyasaponin1 with the enzyme. The hypothesis is that this binding is partly reversed when the enzyme–inhibitor complex formed during the preincubation is diluted with the reaction mixture containing the substrate (indicated by the partial rate recovery). The recovery is more significant at lower concentrations of Soyasaponin 1. The slow association may be characteristic of covalent binding.^36^ The absolute value of the Hill slope (Table S3) is higher when calculated at later time points. As the incubation proceeds, the enzyme activity recovers faster for the lower range of the dose response compared to the higher range, which leads to a steeper slope with increasing the incubation. In addition to the possibility of covalent binding as an acting mechanism, the steep slopes and time dependency of IC_50_ are characteristics of nonspecific (nonpharmacological) inhibition often encountered in drug screening campaigns. Compounds with these characteristics are called pan-assay interference compounds.^37^ To reveal the detailed nature of the inhibition, the use of accurate methods, previously developed to distinguish specific/nonspecific, covalent/noncovalent, reversibility, and interaction complex kinetics^37−40^ may be used. In all of these diagnostic methods, the availability of continuous assays is essential to explore this level of mechanistic understanding.

Here a comparative dose response study of the three GTs’ catalytic performance in response to Soyasaponin1 was done, taking care to use the standardized parameters gained from the UGC platform. These parameters include the concentrations of donors and acceptors in relation to their *K*_m_ values. For all three enzymes, the acceptors were added at concentrations equal to two times their *K*_m_, whereas the donors were used at half their *K*_m_. Details of the fitting are explained in the Supporting Information (Table S4). Under these standardized conditions, ST3GAL1 and FUT1 are the most responsive enzyme to Soyasaponin1 inhibition observed in the pIC_50_ values of 4.50 and 4.28 respectively (i.e., IC_50_ values 31 and 52 μM), respectively. However, C1GALT1 showed a poor response with a pIC_50_ values ≪4.50 (i.e., IC_50_ ≫ 100 μM). Although a comprehensive GT screening study for Soyasaponin1 was not undertaken, these preliminary results lead to the conclusion that the inhibitor is not ST specific. The pIC_50_ of ST3GAL1’s dose response to Soyasaponin1 increased from 4.23 to 4.50 (i.e., an decrease of IC_50_ from 59 to 31 μM) with reduced donor levels (Figure 8B,C), which can be attributed to a competitive mechanism of Soyasaponin1 with the donor as was reported in the original study. However, confirmation of the mechanism of action requires detailed diagnostic tests^41^ that fall out of the scope of this work.

To rule out the possibility of inhibition observed in this trial being exerted on the coupling enzymes CMK, NDK, PK, or LDH, the highest dose used in the study (120 μM) was tested with reaction mixtures of 50 μM UDP, GDP, or CMP with their corresponding coupling substrates and enzymes. The results showed no inhibition for a range of Soyasaponin1 concentrations in any of the assay coupled component enzymes reactions. This confirms that the inhibitory effect observed was effective only on the GTs. The method demonstrated on Soyasaponin1 is a guideline that can be applied in generic high-throughput screening, inhibitory mechanism, and specificity studies.

## 3 Methods

### 3.1 Materials

#### 3.1.1 *E. coli* Cell Culture and Recombinant Protein Expression

*E. coli* BL21(DE3): (Sigma-Aldrich, Cat. no. CMC0014), *E. coli* BL21(DE3): DH5α: cells were a gift from E. Sturrock (University of Cape Town, South Africa). Expression plasmids for NDK and CMK were designed from the full sequences of the enzymes, YP_003986922.1 and WP_000125016.1, respectively, in the pET-21b(+) backbone plasmid. Final constructs pET-21b(+)-NDK-6XHis and pET-21b(+)-CMK-6XHis were synthesized at BIOMATIK (Ontario, Canada). Expression plasmid for TEV protease enzyme pRK793 was a gift from David Waugh (Addgene plasmid #8827; http://n2t.net/addgene:8827; RRID: Addgene_8827), supplied in *E. coli*. BL21(DE3)-RIL cells. Yeast extract (HG000BX6), bacteriological agar (HG000BX1), and tryptone (HG000BX4) were purchased from Biolab (Merck, Modderfontein, South Africa). Ampicillin (A9518), chloramphenicol (C0378), protease inhibitor (cOmplete, 11873580001), lysozyme (10837059001), and isopropyl β-d-thiogalactoside (IPTG, 16758) were obtained from Sigma-Aldrich.

#### 3.1.2 Mammalian Cell Culture and Recombinant Protein Expression of GTs

HEK293F cells (R79007, Thermo Fisher) were a gift from E. Sturrock (University of Cape Town, South Africa). All expression plasmids for GTs used in this work were purchased from the plasmid repository DNASU. These expression vectors are the pGEn2 vectors constructed by Moremen et al. (2018).^42^ A full list of the constructs can be found on the repository database; http://glycoenzymes.ccrc.uga.edu/. Plasmids were propagated and purified using Genejet Plasmid Maxiprep Kit (Thermo Fisher, K0492) following the manufacturer’s protocol. HEK293F Freestyle 293 Expression Medium (Thermo Fisher Scientific, cat. no. 12338026). Polyethylenimine (PEI), linear, MW 25,000, transfection grade (cat. no. 23966-1) was purchased from Polysciences (USA) and valproic acid sodium salt (VPA, P4543) from Sigma-Aldrich.

#### 3.1.3 Assay Reagents and Enzymes

l-Lactic dehydrogenase (LDH, L2500), PK (P1506), cytidine 5′-monophosphate (CMP, C1006), bovine serum albumin (BSA, A3059), *N*-(2-hydroxyethyl) piperazine-*N*′-(2-ethanesulfonic acid), 4-(2-hydroxyethyl) piperazine-1-ethanesulfonic acid (HEPES, H3375), CMP-sialic acid (CMP-Neu5Ac, C1006), uridine 5′-diphosphate disodium (UDP, 94330), phosphoenolpyruvic acid monopotassium salt (PEP-K, 860077), adenosine 5′-triphosphate disodium salt trihydrate (ATP, 10519979001), β-nicotinamide adenine dinucleotide, and reduced disodium salt hydrate (NADH, 10128023001) were all purchased from Sigma-Aldrich. Other substrates, cytidine diphosphate (CDP, NC09380), guanosine diphosphate (GDP, FG152484), galacto-*N*-biose (OA01686), lacto-*N*-biose (OL01707), T-Bn (OB05157), GalNAc-Ser (MA07378), GDP-l-fucose disodium salt (GDP-Fuc, MG01912), UDP-α-d-galactose disodium (UDP-Gal, MU58246), and the inhibitor Soyasaponin1 (FS65454) were all purchased from Biosynth (Carbosynth).

### 3.2 Recombinant Protein Expression and Purification

Expression of GT enzymes was carried out according to the protocol established previously.^42^ Briefly: starting with a healthy cell culture (viability >95%) a subculture in 100 mL of Freestyle media to a cell density of 1 million cell/mL is prepared 24 h before transfection. On the transfection day, media was replaced with 50 mL of fresh media (final cell count after media exchange is 2.5–3 million cell/mL). After 10 min of incubation, 150 μg of plasmid was added. The culture was incubated for an additional 5 min before 450 μg of PEI was added dropwise, equivalent to 900 μL of 0.5 mg/mL PEI, which was made up with 1 mg/mL PEI diluted 1:1 with Freestyle media. The culture was further incubated for 24 h, and then 50 mL of Freestyle media containing 4.4 mM valproic acid was added to achieve a total culture volume of 100 mL containing 2.2 mM valproic acid. Samples of the culture were taken daily and examined under a fluorescence microscope to examine the protein expression and cell viability. The expression was terminated when cell viability fell below 50% and the medium was collected for protein purification. C1GALT1 was coexpressed with its chaperone C1GALT1C1 by adding both plasmids in the transfection step.

Expression of TEV protease was carried out using the protocol published previously.^43^*E. coli* Bl21(DE3) cells were transformed with pET-21b(+)-NDK-6XHis and pET-21b(+)-CMK-6XHis using common heat shock protocols. Expression of NDK and CMK was carried out in Luria Broth supported with ampicillin to a final concentration of 50 μg/mL at 37 °C overnight with shaking at 150 rpm. The induction of expression was done by adding IPTG to a final concentration of 0.5 mM when the optical density of the culture reached (0.4–0.5) after inoculation.

Sample preparation for immobilized-metal affinity chromatography (IMAC) was carried out as follows: for GT enzymes, the media after expression were collected and clarified by filtration (using 0.45 μm sterile filters). For NDK and CMK, the cell pellets from *E. coli* expression were lysated by incubation in 4 °C for 4 h in IMAC binding buffer (20 mM Tris-HCl, 5 mM imidazole-HCl, 500 mM NaCl, 0.05% (w/v) sodium azide, pH 7.9) supporting with 1 tablet of protease inhibitor and 20 mg of lysozyme per 20 mL buffer. Cell lysate was clarified by centrifugation at 48000 RCF for 30 min and filtration by 0.45 μM sterile filter. IMAC was performed using the protein liquid chromatography (FPLC) system ÄKTA Start. The column used for IMAC is a 1 mL HiTrap Chelating High-Performance column (cat. no. 17-0408-01), purchased from Cytiva (USA). Elution of the captured protein was carried out by a gradient from 5 to 500 mM imidazole-HCl over 15 min at a flow rate of 1 mL/min. Elution fractions were collected, and SDS-Page gel was performed to confirm the purity and the size of the proteins. All purified enzymes were quantified using Bradford protein assay kit (Therm oFisher, A55866) and stored at −80 °C in freezing buffer (20 mM Tris-HCl, 150 mM NaCl and, 10% (v/v) Glycerol, pH 7.6).

### 3.3 GT Tag Removal via TEV Protease

GTs expressed from pGEn2 vectors are N-terminally tagged with signaling peptide-8X His-Avi tag-super folder GFP-Tev protease recognition sites. To remove the tag and obtain the enzymes in their native sequence, purified (tagged) proteins were incubated overnight at 4 °C with TEV protease at a ratio of 1:5 (TEV protease: fusion protein) in TEV protease buffer (50 mM Tris-HCl, 0.5 mM EDTA, 1 mM DTT, pH 8.0). The mixture was then processed by an IMAC to collect the untagged enzymes from the flow through fractions. Enzymes were stored at −80 °C in freezing buffer (20 mM Tris-HCl, 150 mM NaCl and 10% (v/v) Glycerol, pH 7.6).

### 3.4 Fluorescence Measurement Optimization

NADH serial dilution was prepared in the assay reaction buffer: 50 mM HEPES–NaOH buffer, 50 mM KCl, 10 mM MnCl_2_, 10 mM MgCl_2_, and 1 mg/mL BSA, 2 mM PEP, 2 mM ATP, pH 7.4. Thirty μL of each dilution was distributed in three replicates into the wells of a 384 well plate (Corning, 4514). Fluorescence was recorded over 30 min using the multiplate reader, EnVision 2102 (PerkinElmer) with the excitation and the emission filters, Photometric 340, Narrow 340 and Umbelliferone 460, respectively. Other parameters were kept at the default setting. Linearity of the signal was detected from a range of NADH concentrations from 0 to 150 μM. Instrument parameters: measurement height, number of flashes, photomultiplier tube gain, and excitation and emission light intensities were adjusted using EnVision’s built-in optimization program to maximize the *Z̀* for a range between 75 and 100 μM of NADH. The result optimized parameters were used throughout the rest of the study.

### 3.5 UGC Coupling Assay

The following optimized parameters are consistent throughout the study, (1) Reaction buffer: 50 mM of KCl, 10 mM of MnCl_2_, 10 mM of MgCl_2_, and 1 mg/mL of BSA; pH adjusted to 7.4 after adding the other components, (2) Enzymes: 6 units of PK and 3 units for LDH (per 30 μL reaction). The unit is defined by the amount of the enzyme that converts 1 μmol substrate to product per minute. This amount may vary from batch to batch. (3) 2 mM PEP, 2 mM ATP, and 0.1 mM NADH (with a tolerance range of 0.05–0.15 mM). (4) NDK and CMK are used (NDK alone for UDP and GDP detection or in combination with CMK for CMP detection) at concentrations of 500 and 390 nM, respectively.

#### 3.5.1 Kinetics Assay

Kinetics assays were performed by preparing master mixtures consisting of all of the reaction components except for the variable component whose concentration effect on the reaction would be tested. Components of the master mixtures were prepared in concentrated solutions to account for the concentration adjustment when all of the components are added. Water was used to make up the volume, as needed. The variable component was distributed to the wells in triplicate and both the master mixture. The variable component was incubated at 37 °C prior to the initiation of the reaction. Incubation was needed to deplete the nucleotides potentially present in the master mixtures (such as ADP from the ATP solution). Initiation was carried out simultaneously by distributing the master mixture to the wells using a multichannel pipet. The fluorescence signal was immediately recorded in 15 s intervals using kinetics mode. The concentrations used in the kinetics studies were as follows:1. ST3GAL1: enzyme concentration at 226 nM, galacto-*N*-biose (0–1 mM, with CMP-Sia fixed at 1 mM), CMP-Sia (0–1 mM, with galacto-*N*-biose fixed at 1 mM). 2. C1GALT1: enzyme concentration at 266 nM, GalNAc-Ser (0–6 mM, with UDP-Gal fixed at 10 mM), UDP-Gal (0–10 mM, with GalNAc-Ser fixed at 3 mM). 3. FUT1: enzyme concentration at 500 nM, T-Bn (0–6 nM, with GDP-Fuc fixed at 0.3 mM), lacto-*N*-biose (0–6 mM, with GDP-Fuc fixed at 0.3 mM). Calculations of the rates and fitting to the Michaelis–Menten model are explained in the supporting data (Figures S4, S5, and Table S2 Supporting Information).

#### 3.5.2 Inhibition Assay

Soyasaponin 1 stock solution was prepared at 1.25 mM in 2% (v/v) Triton X-100 and 10% (v/v) DMSO. Inhibition assays were performed following a method similar to that used in the kinetics study. The master mixtures included all of the components except for the inhibitor and the enzyme. A 2-fold serial dilution from the inhibitor’s stock solution was prepared in a blank solution containing 2 and 10% (v/v) Triton X-100 and DMSO, respectively. The enzyme and the inhibitor were mixed, distributed to the wells, and preincubated for 30 min at 37 °C. A 10 min incubation period was also carried out at 37 °C for the master mixture, and the reaction was initiated by adding the master mixture to the wells. The addition of the reaction mixture resulted in a 5-fold dilution of the enzyme–inhibitor mixture. The inhibition assay included two controls: a full reaction without an inhibitor and a full reaction without an acceptor (and without an inhibitor). In these controls, the enzyme was also incubated for 30 min with a blank solution containing 2 and 10% (v/v) Triton X-100 and DMSO, respectively. The difference in the slopes of the two controls was used to normalize the activity. The effect of the activity percentage was calculated at each concentration of the inhibitor as follows
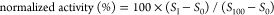
where *S*_I_, *S*_100_, *S*_0_ are the absolute values of the slopes from the progress curves of the reaction with inhibitor, reaction without acceptor, and full reaction (without inhibitor) respectively. IC_50_ and pIC_50_ (−log IC_50_ in molar concentration) values were calculated as depicted in Tables S3 and S4 (Supporting Information). Rates for the comparative dose response were calculated for each enzyme at the last time point where the uninhibited reaction maintained its linearity. These time points are at 2, 5, and 20 min for FUT1, C1GALT1, and ST3GAL1, respectively. Examples of the effect of Soyasaponin1 on the progress curves of FUT1 and C1GALT1 are presented in Figure S6.

### 3.6 Reaction Modeling

The PK/LDH computational simulation was performed using COPASI v.4.4 (build 278) software.

#### 3.6.1 Model Setup

The two reactions were set as follows
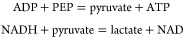


A generic rate law for a reversible Bi–Bi reaction following sequential reactions (single central complex),^44^ was used for both reactions, which is expressed as follows
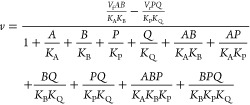
where *A*, *B*, *P* and *Q* were defined as the concentration of ADP, PEP, pyruvate, and ATP for the PK reaction and as NADH, pyruvate, lactate, and NAD for the LDH reaction. *K*_A_, *K*_B_, *K*_P_, *K*_Q_ are Michaelis–Menten constants for the substrates and products. *V*_f_ and *V*_r_ are the maximum velocities for the forward and reverse reaction, respectively. Based on the definition of the unit for PK and LDH, 1 unit of PK and LDH added to 30 μL of a reaction mixture produces a *V*_max_ value of 33.3 mM/min when the enzyme is saturated with its substrates. The maximum forward velocities values *V*_f_ were calculated from the number of units added to the reaction for each of PK and LDH. Vr values were calculated from the relation: *K*_eq_ = *V*_f_*K*_P_*K*_Q_/*V*_r_*K*_A_*K*_B._ The values of *K*_eq_ for PK and LDH were obtained from curated values in literature,^44^*K*_m_ values were obtained from Table S1 (Supporting Information). The calculations of *V*_r_ values for the PK and LDH reactions produced values of 2.12 and 0.028 mM/min, respectively.

#### 3.6.2 Time Course Simulation

The simulation was performed in a time course for initial concentrations of the reaction’s species that are defined in the result section (Section 2.1.2 and Figure 3). Simulation was run for 0.1 min, and concentrations of the species were collected for 40 intervals within the simulation duration. Data were exported and plotted for each ADP concentration in one set (Figure 3). Concentrations of NADH were converted to “Residue percentage” as shown in Figure 3. This was calculated from the formula: residue (%) = 100. (*A* – *A*_t_)/(*A*_0_ – *A*_t_); *A* is the concentration at a given point of time, *A*_t_ is the terminal concentration after complete depletion (equal to NADH initial concentration – ADP initial concentration), *A*_0_ is the initial concentration.

### 3.7 Statistics

All of the data points were repeated three times. Shapiro–Wilk normality tests were performed to confirm the normal distribution of the data. Normality tests and regressions were performed using GraphPad Prism 8 software. The data are presented as (mean value) ± (standard deviation) in all linear regression, kinetics direct, and inhibition dose response plots (except when these bars were not visible because of the near insignificant standard deviations).

## Supporting Information Available

The Supporting Information includes progress curves of the assay optimization, example of kinetics data processing, and the statistics of regression (PDF)

## Abbreviations

ADP: adenosine diphosphate
ATP: adenosine triphosphate
C1GALT1: core 1 β 1,3-galactosyltransferase
CDP: cytidine diphosphate
CMK: cytidylate kinase
CMP: cytidine monophosphate
GalNAc: *N*-acetyl-d-galactosamine
GDP: guanosine diphosphate
GT: glycosyltransferase
LDH: lactate dehydrogenase
NDK: nucleoside diphosphate kinase
NDP: nucleoside diphosphate
PEP: phosphoenolpyruvate
PK: pyruvate kinase
ST3GAL1: sialyltransferase 3, *N*-acetylgalactosaminide α-2,3-sialyltransferase 1
TEV: tobacco etch virus
UDP: uracil diphosphate
