# Supplementary material for: Universal Glycosyltransferase Continuous Assay for Uniform Kinetics and Inhibition Database Development and Mechanistic Studies Illustrated on ST3GAL1, C1GALT1, and FUT1
Source: ACS Omega. 2024 Apr 5;9(15):17518–32. doi: 10.1021/acsomega.4c00485 (PMC11025096; doi:10.1021/acsomega.4c00485)
Supplement: Supplementary file 1 — ao4c00485_si_001.pdf [file ao4c00485_si_001.pdf]

Universal Glycosyltransferase Continuous Assay for Uniform Kinetics and Inhibition Database Development and Mechanistic Studies Illustrated on ST3GAL1, C1GALT1, and FUT1

Abdullateef Nashed[1,2] and Kevin J. Naidoo[1,2*].

[1]Scientific Computing Research Unit, PD Hahn Building, University of Cape Town, Rondebosch 7701, [2]Department of Chemistry, PD Hahn Building, University of Cape Town, Rondebosch 7701.

*To whom correspondence should be addressed

Supporting Information

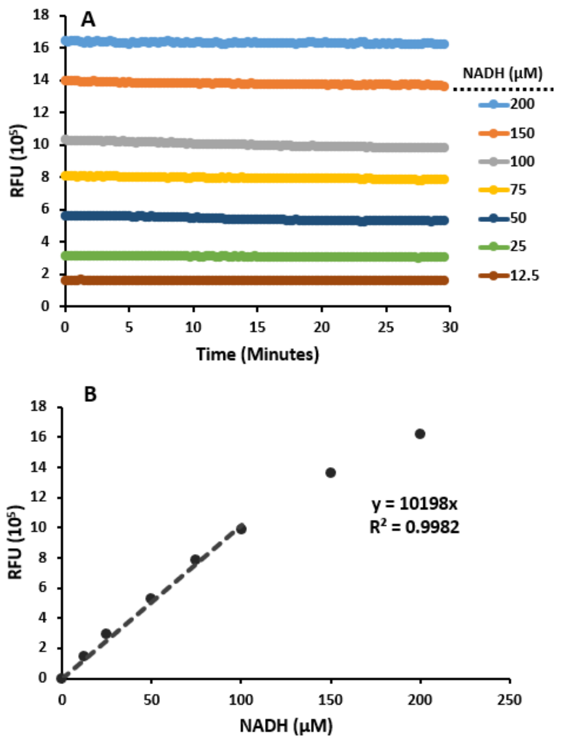

Figure S1. Linearity and stability of NADH fluorescence. A, Stability of fluorescence signals of NADH serial dilution in PK/LDH reaction mix during recording for 30 minutes. B, Means of fluorescence for each concentration during the recording period are plotted against concentration. Linear regression for the range 0-100 μM with the equation and R^2 value are shown. Concentrations were prepared in triplicate.

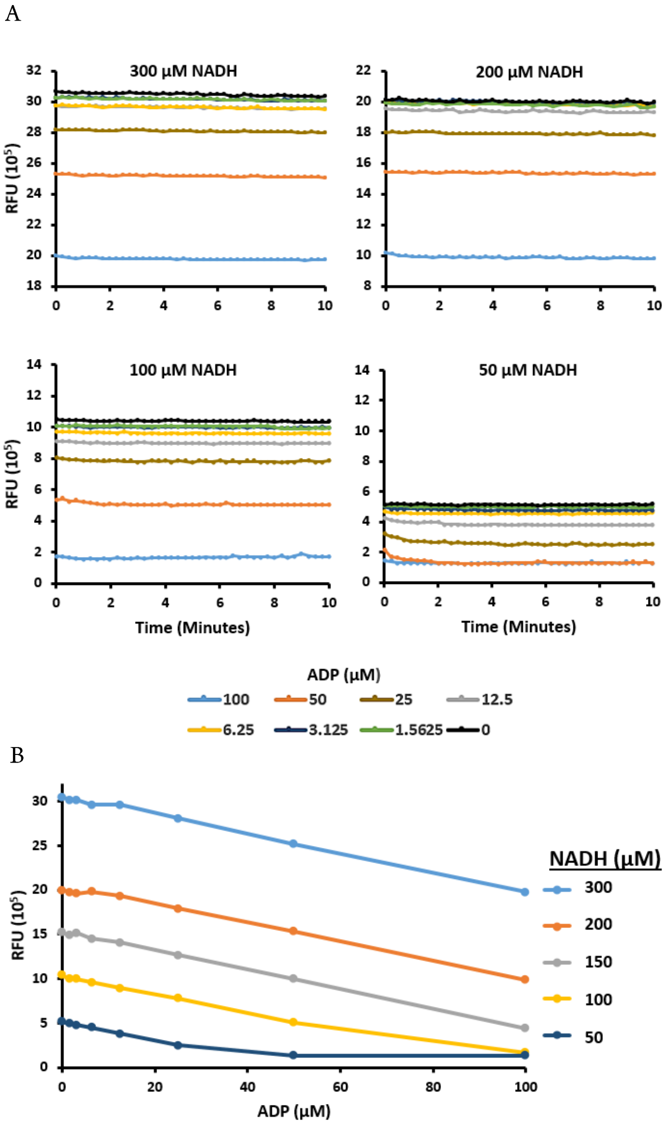

Figure S2. Experimental validation of NADH effect on PK/LDH coupled reaction performance. A, time course of PK/LDH reaction with a twofold serial dilution of ADP was performed at different concentrations of NADH. B, fluorescence vs ADP concentration. Fluorescence values at each concentration of ADP were calculated as the mean of the signal over 10 minutes.

Table S1.

Enzyme	K_M (mM)										
	ATP	ADP	GDP	UDP	CDP	CMP	Pyruvate	PEP	NADH	NAD^+	Lactate
LDH	-	-	-	-	-	-	0.13*	3.50**	0.058**	0.23**	10**
PK	0.86#	0.24** - 0.30#	0.26**	1**	6.8**	-	10#	0.07#	-	-	-
CMK	0.038*	-	-	-	-	0.035*	-	-	-	-	-
NDK	0.005*	0.77*	0.02-0.3**	0.1*	0.05*	-	-	-	-	-	-

Kinetics parameters of the enzymes used in the study for the corresponding substrate. The source of the information presented in the table are as follows:

Supplier

*Media (Brenda for the same species) excluding mutant enzymes.

** Median (Brenda all reported values) from different species, excluding mutant.

(-): values are not available (or values are irrelevant to the study)

The activity of NDK and the effect of its concentration was tested on phosphorylating 50 µM UDP in the optimized PK/LDH reaction mixture (Figure S3). The depletion was instant in all tested concentrations of NDK, whereas there was a lag of 2 minutes in absence of NDK. To further investigate the role of NDK on UDP and GDP phosphorylation, serial dilutions of both nucleotides were reacted with reaction mixtures in presence or absence of 500 nM NDK (Figure 4). The effect of NDK in eliminating the lag time observed in Figure S1 was confirmed for all tested concentrations of UDP. In the case of GDP, the addition of NDK increased the sensitivity of coupled reaction to GDP.

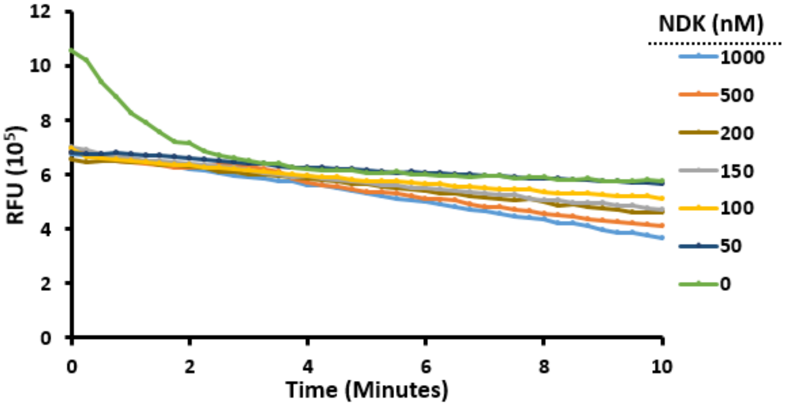

Figure S3. Titration of NDK with 50 µM UDP. A serial dilution of NDK was incubated with PK/LDH reaction mixture contains 50 µM UDP as calculated in the final reaction volume.

Progress Curves

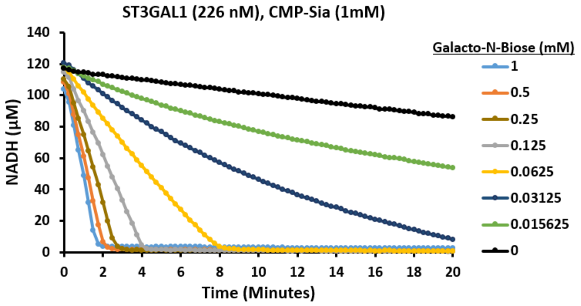

Galacto-N-Biose (0 mM)

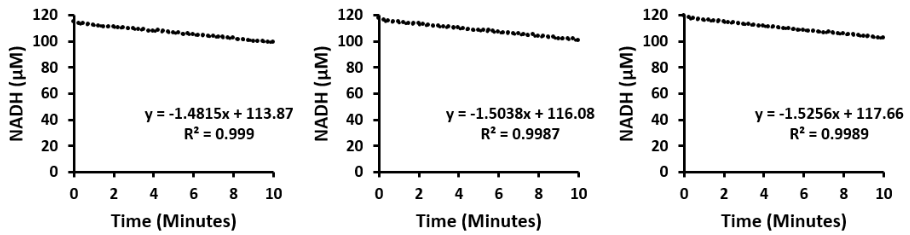

Galacto-N-Biose (1 mM)

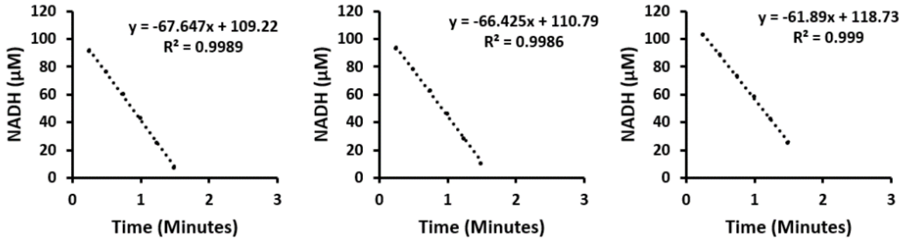

Galacto-N-Biose (0.0156) mM

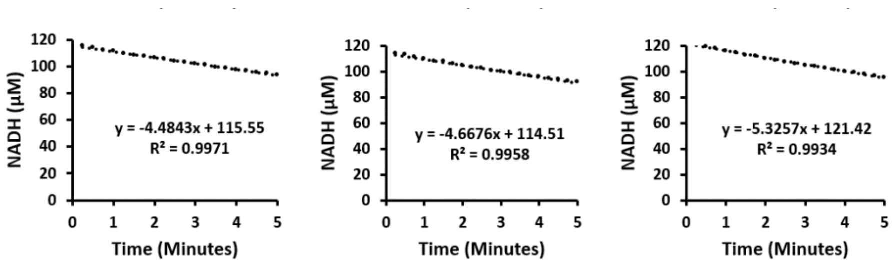

Figure S4. Enzymatic Kinetics of ST3GAL1. Progress curves of the glycosylation of Galacto-N-biose by ST3GAL1 at a fixed concentration of the donor CMP-Sia of 1 mM and varied concentrations of Galacto-N-Biose are shown in the top panel. fluorescence signal was converted to NADH concentration from NADH standard curve (Figure S1). Progress curves shown in the top panel are the means of three replicates carried out for each concentration of Galacto-N-Biose. Detailed linear regression demonstrated for the three replicates carried out at three concentrations of Galacto-N-Biose are shown. Regression was performed on the linear range to avoid the lag phase (pre-steady state) and rate decline due to substrate consumption. The slopes calculated from the linear regression represent NADH depletion rate in µM/min.

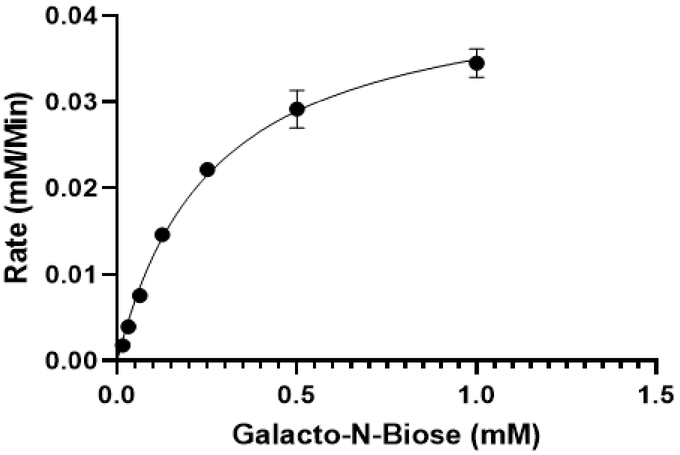

k_{cat} Best-fit values	
Et (mM)	= 0.0002263
k_{cat}	195
K_m (mM)	= 0.2633
V_{max} (mM/Min)	= 0.04414
**95% CI (profile likelihood)**	
k_{cat}	189.2 to 200.9
**Goodness of Fit**	
Degrees of Freedom	6
R squared	0.9974
Sum of Squares	2.56E-06
Sy.x	0.000653
**Constraints**	
Et (mM)	Et = 0.0002263
K_m (mM)	K_m = 0.2633
**Number of points**	
# of X values	7
# Y values analyzed	7

Figure S5. Michaelis-Menten kinetics of ST3GAL1. CMP release rate was calculated from The NADH depletion rates calculated from the progress curves shown in Figure S4 by dividing these rates by 1.9, which is the stoichiometric ratio of released CMP to oxidized NADH in the CMK/NDK/PK/LDH coupling assay format (Figure 5, F). The fitting of the reaction rates vs concentration of Galacto-N-Biose to Michaelis-Menten model was performed using GraphPad Prism8 software. Et is the enzyme concentration. The rates are shown as means ± SD, n = 3. Distribution normality was tested using Shapiro-Wilk test. Standard deviation bars for some points are too small to be visible in the figures.

Lacto-N-biose Michaelis-Menten Best-fit values	
V_{max} (mM/Min)	0.1784
K_m (mM)	15.1
**95% CI (profile likelihood)**	
V_{max} (mM/Min)	0.1215 to 0.3628
K_m (mM)	8.885 to 35.73
**Goodness of Fit**	
Degrees of Freedom	5
R squared	0.997
Sum of Squares	6.305E-06
Sy.x	0.001123
**Constraints**	
K_m	$K_m > 0$
**Number of points**	
# of X values	7
# Y values analyzed	7

T-Bn Michaelis-Menten Best-fit values	
V_{max} (mM/Min)	0.07165
K_m (mM)	12.25
**95% CI (profile likelihood)**	
V_{max} (mM/Min)	0.05387 to 0.1104
K_m (mM)	8.140 to 21.41
**Goodness of Fit**	
Degrees of Freedom	5
R squared	0.9978
Sum of Squares	9.65E-07
Sy.x	0.000439
**Constraints**	
K_m	$K_m > 0$
**Number of points**	
# of X values	7
# Y values analyzed	7

Table S2. Michaelis-Menten kinetics fitting of FUT1 direct plot. The fitting of the reaction rates vs concentration of Lacto-N-Biose and T-Bn to Michaelis-Menten model was performed using GraphPad Prism8 software.

	5 Minutes	10 Minutes	20 Minutes
**log [Inhibitor] vs. normalized response -- Variable slope**			
**Best-fit values**			
**Log IC50 (M)**	-4.615	-4.345	-4.231
**Hill Slope**	-1.985	-2.403	-3.106
**IC50 (M)**	2.428×10^{-5}	4.519×10^{-5}	5.872×10^{-5}
**Std. Error**			
**Log IC50 (M)**	0.05262	0.03775	0.01551
**Hill Slope**	0.3648	0.4193	0.3258
**95% CI (profile likelihood)**			
**Log IC50 (M)**	-4.769 to -4.482	-4.461 to -4.253	-4.273 to -4.195
**Hill Slope**	-3.812 to -1.260	-4.239 to -1.496	-4.048 to -2.395
**IC50**	1.703×10^{-5} to 3.294×10^{-5}	3.462×10-5 to 5.582×10^{-5}	5.332×10^{-5} to 6.383×10^{-5}
**Goodness of Fit**			
**Degrees of Freedom**	7	7	7
**R squared**	0.9539	0.9616	0.9893
**Sum of Squares**	541.3	429.5	124.9
**Sy.x**	8.793	7.833	4.224
**Number of points**			
# of X values	9	9	9
# Y values analyzed	9	9	9

Table S3. Inhibition dose response fitting at variable time points of the progress curves of ST3GAL1 inhibition by Soyasaponin 1 (Figure 8, B). Normalized activity % values were calculated from the slopes of the progress curves at different concentrations of Soyasaponin 1 presented in Figure 8, A, and explained in the method section. The relationship of normalized activity values and inhibitor concentrations (transformed to log values in molar concentration) at each time point were fitted to Log [Inhibitor] vs. normalized response-Variable slope model on GraphPad Prism8 software.

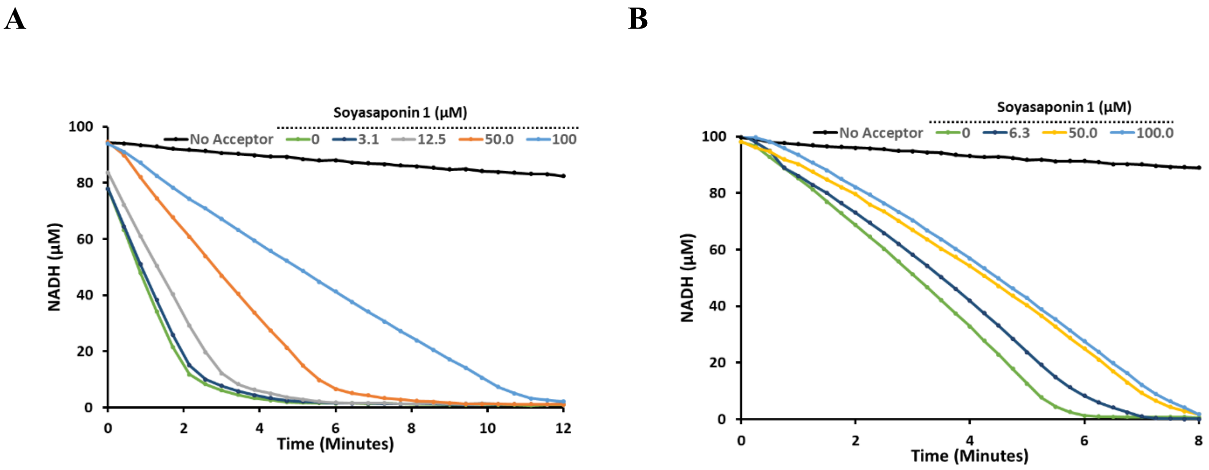

Figure S6. Progress curves showing Soyasaponin1 inhibition on FUT1 (A) and C1GALT1 (B) enzymatic reactions with concentrations of the donors equal to half their K_m, and for the acceptors equals to double their K_m. A control reaction without acceptor was included. Details of the procedure are explained in the method section. Calculations of rates and dose response fitting are in table S4 and method section in the main text.

	FUT1	C1GALT1	ST3GAL1
**log [Inhibitor] vs. normalized response -- Variable slope**			
**Best-fit values**			
**Log IC50 (M)**	-4.283	-3.435	-4.508
**Hill Slope**	-1.253	-0.4567	-1.853
**IC50 (M)**	5.208×10^{-5}	36.75×10^{-5}	3.104×10^{-5}
**Std. Error**			
**Log IC50 (M)**	0.03538	0.2309	0.05151
**Hill Slope**	0.1462	0.09132	0.3648
**95% CI (profile likelihood)**			
**Log IC50 (M)**	-4.371 to -4.186	-3.838 to -2.507	-4.668 to -4.383
**Hill Slope**	-1.732 to -0.9032	-0.7222 to -0.2646	-3.327 to -1.047
**IC50**	4.260×10^{-5} to 6.518×10^{-5}	14.52×10^{-5} to 311.1×10^{-5}	2.150×10^{-5} to 4.142×10^{-5}
**Goodness of Fit**			
**Degrees of Freedom**	5	5	6
**R squared**	0.9812	0.9037	0.9584
**Sum of Squares**	80.97	92.22	411.5
**Sy.x**	4.024	4.295	8.282
**Number of points**			
# of X values	8	8	8
# Y values analyzed	7	7	8

Table S4. Inhibition dose response fitting for progress curves of FUT1, C1GALT1, and ST3GAL1 inhibition by Soyasaponin 1 (Figure 8, C and Figure S6). Normalized activity % values were calculated from the slopes of the progress curves for each enzyme as explained in method section. The relationship of normalized activity values and inhibitor concentrations (transformed to log values in molar concentration) for each enzyme were fitted to Log [Inhibitor] vs. normalized response-Variable slope model on GraphPad Prism8 software.
